# A New Methodology for 3D Target Detection in Automotive Radar Applications

**DOI:** 10.3390/s16050614

**Published:** 2016-04-29

**Authors:** Fabio Baselice, Giampaolo Ferraioli, Sergyi Lukin, Gianfranco Matuozzo, Vito Pascazio, Gilda Schirinzi

**Affiliations:** 1 Dipartimento di Ingegneria, Università degli Studi di Napoli “Parthenope”, Centro Direzionale di Napoli, Is. C4, Naples 80143, Italy; sergyi.lukin@uniparthenope.it (S.L.); gianfranco.matuozzo@uniparthenope.it (G.M.); vito.pascazio@uniparthenope.it (V.P.); gilda.schirinzi@uniparthenope.it (G.S.); 2 Dipartimento di Scienze e Tecnologie, Università degli Studi di Napoli “Parthenope”, Centro Direzionale di Napoli, Is. C4, Naples 80143, Italy; giampaolo.ferraioli@uniparthenope.it

**Keywords:** Driver Assistance Systems, imaging radar, Compressive Sensing, 3D focus, in depth focus

## Abstract

Today there is a growing interest in automotive sensor monitoring systems. One of the main challenges is to make them an effective and valuable aid in dangerous situations, improving transportation safety. The main limitation of visual aid systems is that they do not produce accurate results in critical visibility conditions, such as in presence of rain, fog or smoke. Radar systems can greatly help in overcoming such limitations. In particular, imaging radar is gaining interest in the framework of Driver Assistance Systems (DAS). In this manuscript, a new methodology able to reconstruct the 3D imaged scene and to detect the presence of multiple targets within each line of sight is proposed. The technique is based on the use of Compressive Sensing (CS) theory and produces the estimation of multiple targets for each line of sight, their range distance and their reflectivities. Moreover, a fast approach for 2D focus based on the FFT algorithm is proposed. After the description of the proposed methodology, different simulated case studies are reported in order to evaluate the performances of the proposed approach.

## 1. Introduction

Passenger vehicle sales have become a growing part of the worldwide economy, with an increasing trend over the last years. With that growth and the advancement of automation technology consumers, governments and society are all demanding better safety and reduction of the amount of deaths and injuries on the roads. Car manufacturers have started implementing Driver Assistance Systems (DAS) in production models as an answer to such demands. Among those, we recall stability control systems, anti-collision systems, Antilock Braking System (ABS), traction control and Electronic Brakeforce Distribution (EBD), seat belts, airbags, shock-absorbing bumpers, anti-intrusion bars, visual systems (VDAS) [1,2]. Most VDAS employ video cameras. They are often used as car parking systems, front or rear and lane vision systems [3,4,5]. Video cameras need outside light sources: the sensor does not work in low visibility or in adverse weather conditions (e.g., fog, rain) and in presence of smoke. These limitations can be overcome by radar technology [6]. Radar-based systems can detect targets hundreds of meters ahead, being minimally affected by fog or heavy rain, *i.e.*, conditions that greatly limit the driver’s field of vision [7]. Radar systems adopt several sensing and processing methods for determining the position and speed of the vehicles ahead [8,9,10]. Usually car manufacturers are very reluctant to alter the shape of the vehicles to accommodate any sensors, so designers are forced to design systems small enough to be mounted inside car’s front grille. In order to combine small dimensions and versatility, small antennas are required, and consequently signals at high frequencies are adopted. In particular, several proposed systems work at 76–77 GHz, which is a good compromise between compactness and cost. In order to produce a high resolution radar imaging system for automotive applications many aspects need to be taken into account, for instance the choice of the more appropriate radar imaging system, the choice of the antenna, the development of a radar and system simulator that helps in evaluating system performance by generating a controllable synthetic environment and, once obtained the image, the post-processing stage, such as target detection and tracking.

At present, several technological solutions for automotive radar imaging systems have been developed. The systems synthesize, analogically or digitally [6], a beam scanning the area of interest to identify targets. The analog synthesis and scanning of the beam can be obtained in several ways, such as phased arrays, travelling wave antennas, and lens antennas. The best performing architecture in terms of resolution and scanning range is a phased array. This solution is also the most expensive, so it is necessary to find a compromise between price and performance. One possibility is to adequately process the signals of several antennas to synthesize a larger array [7]. Another possibility is to use a modular architecture by splitting the array into identical sub-arrays, the feed of each of which is individually controlled.

In the post-processing stage, the radars currently used in the automotive industry are based on the Ultra-wide Band structure [6,10]. Most of the algorithms, developed with the aim of cleaning/reducing noise in the data and classifying targets, are based on the statistical analysis of backscattered radar returns, followed by a statistical classification which allows individuating the category to which the observed target pertains. Both for detection and classification, statistical models are fitted to the data in order to assess their suitability and either confirm or reject the membership of a target to the proposed class.

In this manuscript, we focus on the signal processing step. In particular, we propose a novel signal processing algorithm, based on Compressive Sensing (CS) theory [11,12], for the detection and 3D imaging of targets within an observed volume, even in the case of scatterers sharing the same line of sight (LoS). This goal is achieved by detecting the presence of targets in the observed scene, estimating their positions and inferring their reflectivities. It will be shown that, by exploiting the solution sparsity property, CS techniques result particularly effective in solving such detection and estimation problems. The following of the paper is divided into four sections: the description of the acquisition model is made in Section 2. The CS-based approach is presented in Section 3. Results on simulated data are reported in Section 4. Conclusions are drawn in the final section.

## 2. Methodology

**Figure 1:**
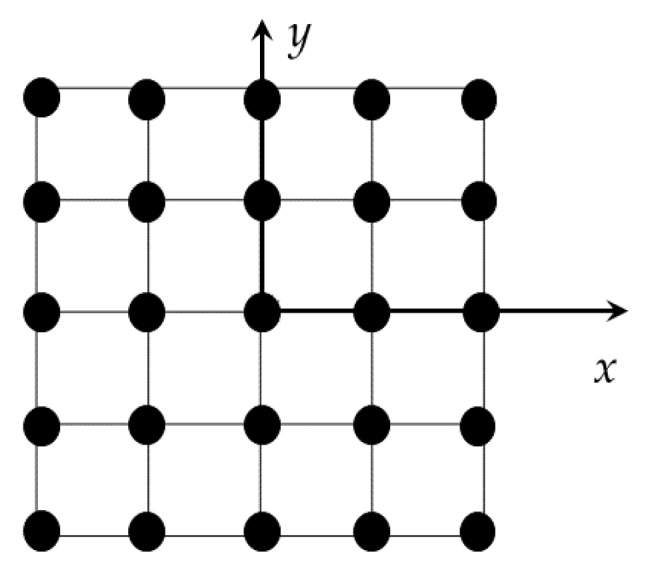
Planar antenna and Cartesian reference system *x*, *y* with origin in the center of the antenna. Black dots highlight the positions of antenna elements *(x_i_*, *y_j_*).

Let us consider an antenna ideally located at the front of the car, laying in the (*x*, *y*) plane. Let us consider a planar array antenna of *K_N_* × *K_M_* elements, where each one transmits and receives the signal. Let us denote with *x_i_*, *i* = [1, …, *K_N_*] and *y_j_*, *j* = [1, …, *K_M_*], *z* = 0 the coordinates of antenna elements. The schematic view of the antenna is shown in Figure 1.

We assume the transmission of a monochromatic signal *S_T_* at frequency *f*_0_. In the noise free-case and neglecting constants, the signal received by the antenna at the position *(x_i_*, *y_j_*) can be modeled as [13]:

(1) $$S_{R}\left(x_{i},y_{j}\right)={∭}_{V}\gamma \left(x,y,z\right)\frac{G}{{\left[R\left(x_{i}-x,y_{j}-y,z\right)\right]}^{2}}\cdot exp\left(i4\pi \frac{f_{0}}{c}R\left(x_{i}-x,y_{j}-y,z\right)\right)dxdydz$$

where *G* is the antenna gain, *c* is the speed of light and *R*(*x_i_* − *x*, *y_j_* − *y*, *z*) is the distance between each antenna element, with coordinates (*x_i_*, *y_j_*, 0), and a target placed in (*x*, *y*, *z*) with reflectivity γ(*x*, *y*, *z*). The signal *S_R_* coherently collects all the echoes from the illuminated volume *V*, with proper attenuation and phase. This model assumes that all targets are point scatterers, that there is no multipath effect and that the superposition principle stands. Being a good trade-off between complexity and handling, such assumptions are widely adopted.

**Figure 2:**
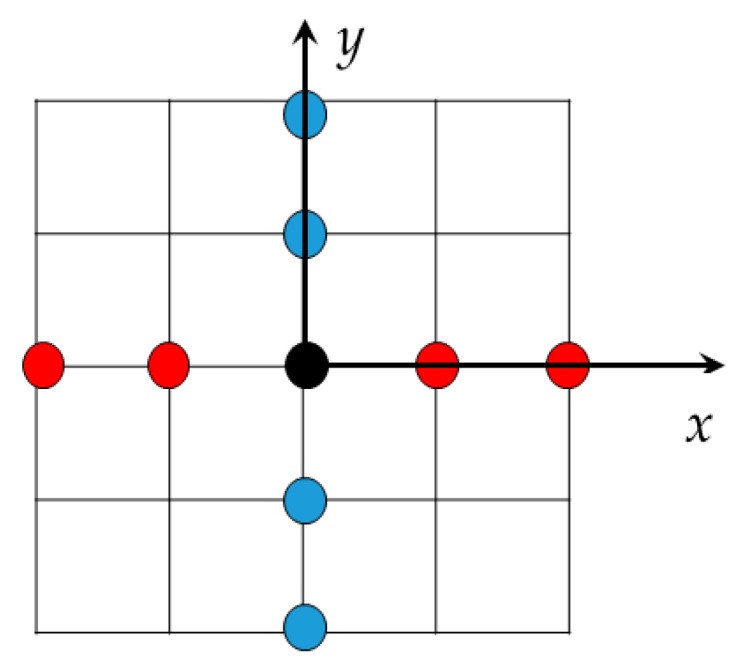
Two linear arrays in cross configuration that synthetize a planar antenna. In this case *K_N_* + *K_M_* elements are considered instead of *K_N_* × *K_M_*.

In order to simplify the realization of the system, the planar antenna is synthetized with the combination of two linear arrays, one horizontal and one vertical, as shown in Figure 2. In this case, the vertical array contains the transmitting elements (blue dots in Figure 2), and the horizontal array contains the receiving elements (red dots in Figure 2).

The vertical array is composed of *K_N_* transmitting elements while the horizontal one is composed of *K_M_* receiving elements. In this case *K_N_* + *K_M_* elements are considered instead of *K_N_* × *K_M_*. The spacing among elements is λ/2. Each transmitting element of the vertical array emits a signal at different time intervals, and each echo is received by all elements of the array of receiving antennas on separate channels. In this case, the distance *R* of Equation (1) can be decomposed as the sum of *R_T_* and *R_R_*, *i.e.*, the distance between the transmitting element and the target and the distance between the target and the receiving element, respectively.

Our aim is to estimate the 3D distribution of scatterers across the imaged volume. In order to speed up the process, only directions, *i.e.*, Lines of Sight (LoS), with the presence of at least one target are selected. To do this, a first processing step implementing a fast 2D focusing algorithm is performed. The goal consists in focusing the acquired signal on a vertical plane at a fixed distance *z_o_*. Developing in Taylor series and truncating at the 2nd term, *R_T_* and *R_R_* can be written as:

(2) $$R_{T}\approx z_{0}+\frac{x^{2}}{2z_{0}}+\frac{{\left(y_{j}-y\right)}^{2}}{2z_{0}}\text{ and }R_{R}\approx z_{0}+\frac{y^{2}}{2z_{0}}+\frac{{\left(x_{i}-x\right)}^{2}}{2z_{0}}$$

Substituting in Equation (1), we obtain:

(3) $$S_{R}\left(x_{i},y_{j}\right)=\iint^{\text{​}}\gamma \left(x,y,z_{0}\right)\frac{G}{{\left[R\left(x_{i}-x.y_{j}-y,z_{0}\right)\right]}^{2}}\exp\left(i\theta \right)exp\left(i\frac{2\pi }{\lambda }\left[\frac{{\left(y_{j}-y\right)}^{2}}{2z_{0}}+\frac{{\left(x_{i}-x\right)}^{2}}{2z_{0}}\right]\right)dxdy$$

where the substitution exp(iθ) = $exp\left(\frac{i2\pi }{\lambda }\left[2z_{0}+\frac{x^{2}+y^{2}}{2z_{0}}\right]\right)$ has been done.

After applying deramping, *i.e.*, correcting the phase term in order to remove the linear component due to the distance, the term exp(iθ) is converted to exp(iϕ), obtaining the signal *S_dr_*:

(4) $$S_{dr}\left(x_{i},y_{j}\right)=\iint^{\text{​}}\gamma \left(x,y,z_{0}\right)\exp\left(i\phi \right)exp\left(-\frac{i2\pi }{\lambda }\frac{xx_{i}}{z_{0}}\right)exp\left(-\frac{i2\pi }{\lambda }\frac{yy_{j}}{z_{0}}\right)dxdy$$

where the intermediate derivations are reported in Appendix.

Moving to a multi-frequency system, the dependency of the acquired signal with respect to frequency has to be made explicit, obtaining *S_dr_*(*x_i_*, *y_j_*, *f*). By assuming a stepped system, a discrete number of frequencies is exploited, thus the vector ***f*** = [*f*_0_, *f*_1_, *f*_2_, *…*, *f_N_*] containing the *N* frequencies can defined.

After discretization, Equation (4) becomes similar to the direct 2D Discrete Fourier Transform expression, with the two exponential functions being the transformation kernel. Thus, the Inverse Fast Fourier Transform (IFFT) algorithm is adopted in order to invert Equation (4) and estimate the term γ(*x*, *y*, *z*_0_) from the acquired signal, *i.e.*,:

(5) $$\gamma \left(x,y,z_{0}\right)=FT^{-1}\left\{S_{dr}\left(x,y,f\right)\right\}\exp\left(-i\phi \right)$$

Note that the data at each considered frequency *f_i_* produces a 2D image of the reflectivity γ(*x*, *y*, *z*_0_). The reflectivity estimated from Equation (5) is exploited in order to detect the presence of targets within the imaged volume and their horizontal θ and vertical φ angles of view.

**Figure 3:**
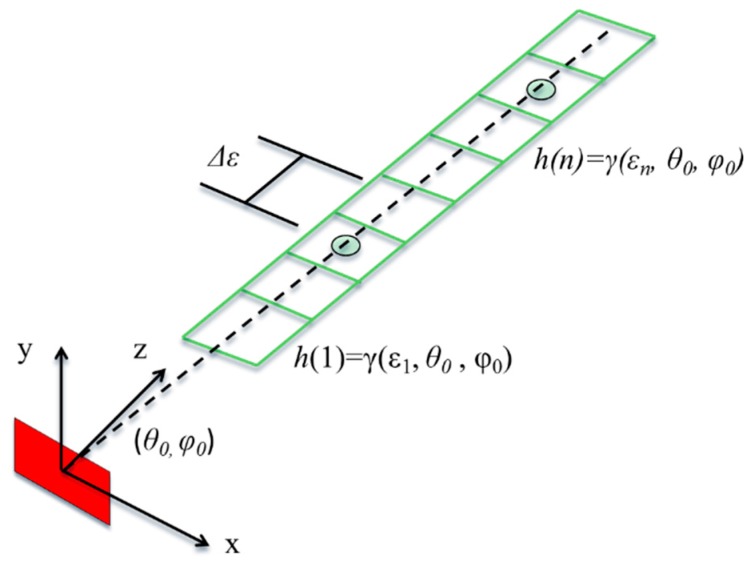
Unknown vector definition: vector *h* contains the reflectivity values at equally spaced range distances in the interval [ε*_min_*, ε*_max_*], for the line of sight (θ_0_, φ_0_).

After this first processing step, a second one is implemented in order to detect the presence of scatterers within the 3D imaged volume and estimate their coordinates. For each identified target (*i.e.*, for each (θ, φ) couple of interest), the antenna beam is tilted to its direction by applying a proper phase correction term to the acquired signals. In this step, we move to the spherical coordinates system (ε, θ, φ) from the Cartesian one (*x*, *y*, *z*). In other words, while 2D focusing works on vertical (*x*, *y*) planes, the 3D focusing considers a volume that is a cone with the vertex positioned in the center of the antenna. Considering the multi-frequency approach, a single complex value is obtained for each of the *N* working frequency. Our aim is, once focused on a LoS, to detect one or multiple targets and estimate their range distances, based on *N* acquisitions. The acquisition model can be written as:

(6) q=Ah

where *q* is the *N* × 1 data vector collecting the focused signals at the different frequencies for the direction (θ, φ), *A* is the transformation matrix and *h* is a vector of the reflectivity at different distances. In particular, the vector *h* contains the complex reflectivity values for different range distances ε_κ_, uniformly sampled in the interval [ε*_min_*, ε*_max_*], as reported in Figure 3. Few targets are expected to be detected for each line of sight, thus most of the *h* elements are supposed to be equal to zero. In other words, *h* can be assumed to be a sparse vector.

Concerning matrix *A*, it is defined by discretizing the acquisition model of Equation (1). The generic element of matrix *A* is:

(7) $$a_{i,j}=\frac{1}{\varepsilon_{j}^{2}}\cdot exp\left(i4\pi \frac{f_{i}}{c}\varepsilon_{j}\right)$$

where *f_i_* is one of the frequencies within the bandwidth and ε*_j_* is a discretized range distance.

Given the previously reported model, our aim is to estimate the number of non-zero elements of *h*, *i.e.*, how many targets are present in the selected line of sight, their position within vector *h*, *i.e.*, the range distances of the detected targets, and their values, *i.e.*, the reflectivity of the targets. We can refer to the estimation of vector *h* as an “in depth” focusing.

In the realistic case, measurements are corrupted by noise, leading to:

(8) q=Ah+w

where *w* is the thermal noise vector, whose element are circular complex Gaussian distributed.

The problem of reconstructing a sparse vector from a low number of measurements is the typical problem addressed by the CS technique. The estimation algorithm can be formulated by solving the following minimization problem:

(9) $$\arg\underset{h}{\min}{||h||}_{1}+\psi \;{||Ah-q||}_{2}$$

where the L^1^-norm promotes the sparsity of the unknown *h* vector, while the L^2^-norm minimizes the difference between the model and the acquired data. ψ is a regularization factor whose ideal value depend mostly on SNR and has to be set [14,15]. In order to compute the estimation of *h*, *i.e.*, the solution of Equation (8), several algorithms can be adopted [11,12,16].

## 3. Results

In order to evaluate the performances of the proposed method, different simulated case studies have been implemented. We simulated, in Matlab^®^ environment, the received signal in the case of difference scenarios, corrupting data with circular complex Gaussian distributed random noise. A cross antenna composed of two linear arrays of 111 (horizontal, Receiving-Rx) and 141 (vertical, Transmitting-Tx) elements has been considered. The system band, between 77 GHz and 77.5 GHz, has been sampled following a stepped approach. Complete system details are reported in Table 1. For the reported simulations, a constant SNR of 30 dB for a target at a distance of 200 m from the antenna has been adopted. In this case, the regularization factor ψ has been empirically set equal to 0.1.

**Figure 4:**
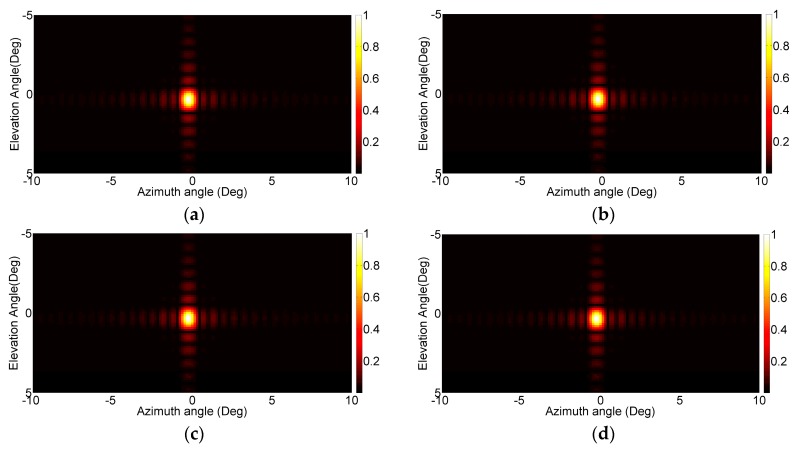
Results of 2D FFT based focusing algorithm in case of *z*_0_ = 10 m (**a**); *z*_0_ = 20 m (**b**); *z*_0_ = 30 m (**c**) and *z*_0_ = 50 m (**d**).

The first dataset is composed of two targets in front of the antenna, *i.e.*, (θ, φ) = (0, 0), with the same reflectivity and range distances of 20 and 30 m, respectively. In Figure 4, the images obtained by the 2D FFT based focus approach, *i.e.*, the first step of the proposed method, considering different distances (*z*_0_) are reported. In particular, 10, 20, 30 and 50 m have been considered. It can be seen that targets are evident in all focusing range distance cases, although at 10 m and 50 m no targets are present, suggesting that the approximations made in Equation (4) hold also in case of wrong range distance assumption. From Figure 4, it can be stated that the choice of the focusing distance *z*_0_ does not noticeably modify the focused image, thus the parameter *z*_0_ can be *a priori* fixed to any value.

Subsequently, we considered the line of sight corresponding to (θ, φ) = (0, 0) for computing the in depth focusing, *i.e.*, estimating the number of targets, their range distances and their reflectivities in front of antenna. Several test studies have been considered in case of different steps in sampling the available bandwidth (in the 77–77.5 GHz interval). In particular, instead of uniformly sampling the bandwidth, random frequencies have been chosen. We first investigated the number of frequencies that has to be considered in order to achieve effective results. For this simulation 500, 100, 20 and 10 frequencies, randomly sampled within the 77–77.5 GHz interval, have been considered. We recall that, the lower the number of adopted frequencies is, the lower the global acquisition time of the system is.

The unknown vector *h* has been assumed to cover a range distance between 10 to 100 m with a spacing of 9 cm, providing 1000 positions. Note that the number or rows of transformation matrix *A* is sensibly lower than the number of columns. In particular, it has 1000 columns and 500, 100, 20 or 10 rows.

**Figure 5:**
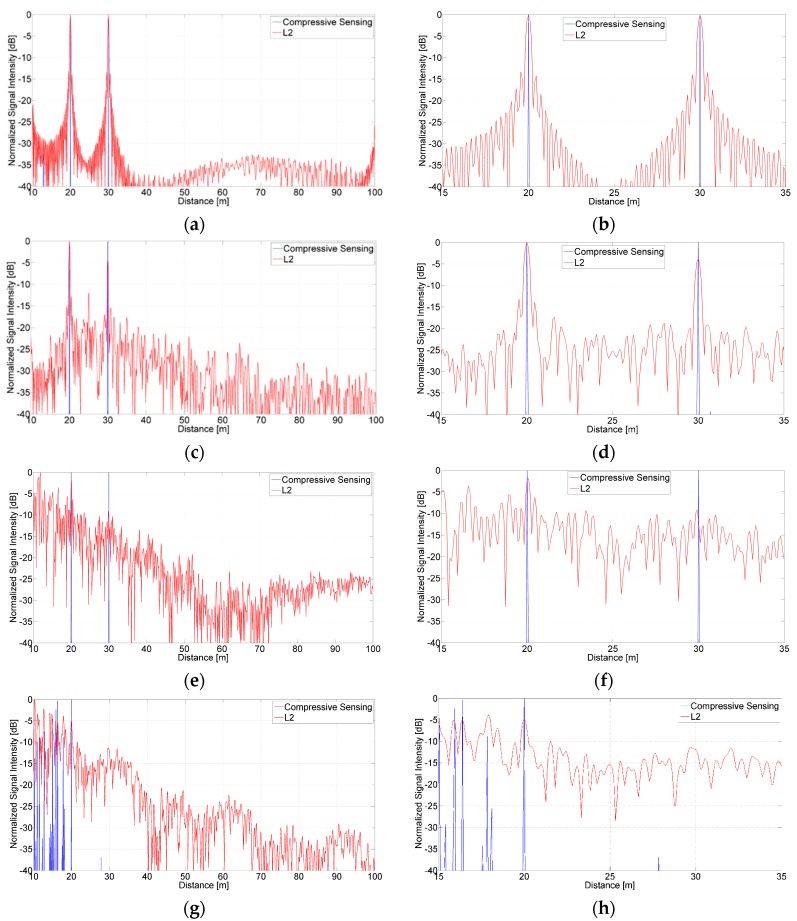
In depth focusing results, with 500 (**a**); 100 (**c**); 20 (**e**) and 10 (**g**) frequencies sampled in the 500 MHz bandwidth. Enlargements related to the 15–35 m range are reported in (**b**,**d**,**f**,**h**). Results of proposed technique (blue line) are compared with the L^2^-norm ones (red line). The target range distances are 20 and 30 m, respectively.

In order to provide a reference solution, the estimation via L^2^-norm minimization technique has also been performed. In Figure 5, results for the L^2^-norm and the proposed CS techniques are reported in red and blue color, respectively, in case of 500 frequencies (Figure 5a), 100 frequencies (Figure 5c), 20 frequencies (Figure 5e) and 10 frequencies (Figure 5g). Each line represents the estimated reflectivity for each range distance between 10 and 100 m. In particular, its value is expected to be zero where no targets are present, while a peak is associated to each detected target within the considered line of sight. In order to better appreciate the results, enlargements in the 15–35 m range are presented for all the cases in the right column of Figure 5.

In case of 500 frequencies (Figure 5a,b), both techniques are able to detect the presence of different targets, their reflectivities and distances (two peaks at 20 and 30 m are evident), with the L^2^-norm approach characterized by a coarser resolution with respect to proposed CS based methodology, as the impulses are larger. Moving to the 100 frequencies case (Figure 5c,d), the proposed approach produces very similar results compared to the previous case, while the L^2^-norm technique shows a much higher amount of estimation noise and fails in evaluating the reflectivity of the target at 30 m. In the 20 frequencies case (Figure 5e,f), characterized by a deeply undersampled bandwidth, the L^2^-norm fails to detect the second target, and shows several false alarms in the 10–30 m range, while the proposed approach still correctly retrieves the scatterers. In the last case, *i.e.*, 10 frequencies sampled within the 500 MHz interval (Figure 5g,h), both techniques fail, as CS is also unable to detect the correct number of scatterers and their distance from the antenna.

**Figure 6:**
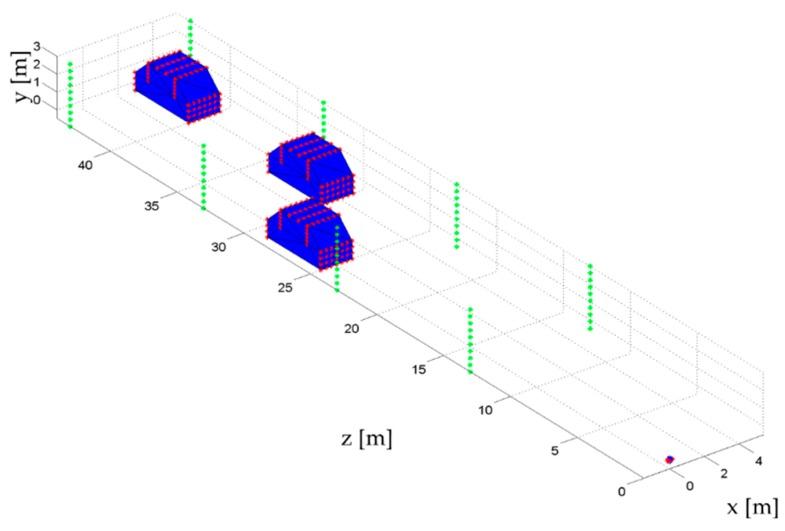
Realistic simulated scenario. Scatterers are positioned in order to simulate a road with cars (red points) and lampposts (green points). Car volume has been marked in blue.

The second simulated dataset is a more realistic scenario. Several scatterers have been placed within the volume of interest in order to simulate a road with cars and lampposts, providing the scenario illustrated in Figure 6. In this case, 100 frequencies have been considered.

**Figure 7:**
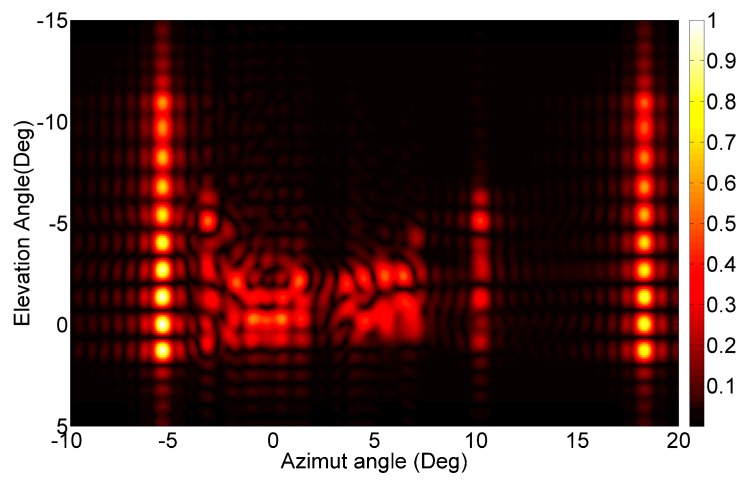
The 2D focusing result for the real scenario, with focusing distance equal to 10 m.

First, the 2D FFT based focus has been applied, providing the image reported in Figure 7. It can be noted that the shapes have been well retrieved, with the lampposts visible on the left and right sides of the images and the cars visible in the central area.

**Figure 8:**
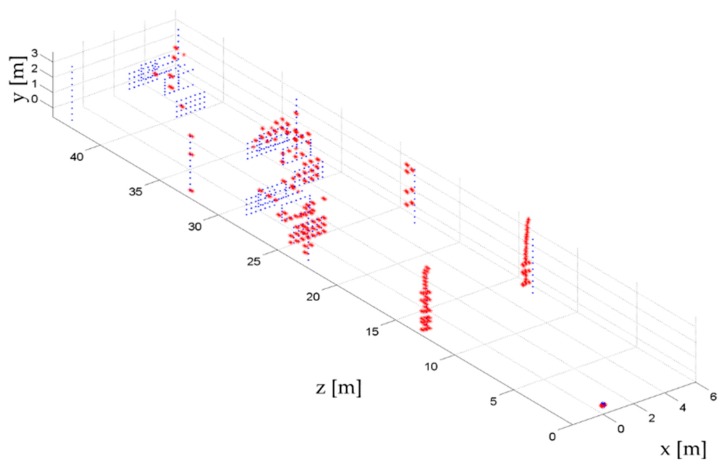
The 3D reconstruction of the realistic scenario. Blue dots represent reference target positions, whereas the red dots are the estimated targets.

The in depth focus via CS approach has been applied for each line of sight, providing the detection of targets within the considered 3D volume. In this case, the L^2^-norm technique provided very unsatisfactory results, thus they have not been reported. In Figure 8 the estimated scatterers (red dots) have been plotted overlapped with the reference scenario (blue dots).

From the reported results, different aspects can be highlighted. The proposed method is able to detect multiple targets sharing the same LoS. The estimated position of the targets is globally satisfactory, *i.e.*, red dots are correctly positioned in the 3D volume. The false alarm rate is very low, even at far range distances from the antenna. Concerning the detection rates, as expected, performances are better in the short range region. From Figure 8 it is evident that the number of detected targets beyond 35 m is very low compared to the nearest region. We have to underline that the considered scenario is very challenging,since in many lines of sight more than two scatterers are present. However, at least few scatterers have been found for each car, while, considering lampposts, only the most distant on the left side of the road has been completely missed.

An evaluation of computation time of the method has been made. At present, the detection of the targets for each line of sight requires about 10 s on a Core i7 workstation with 16 GB of RAM in the case of 20 frequencies and 1000 unknowns. In case of the simulated scenario reported in Figure 8, 119 lines of sight have been detected, thus the simulation was completed in about 119 × 10 s, which is about 20 min in total. However, it has to be underlined that all the process has been implemented in a Matlab environment and no optimization was done on the code. If we accept a resolution reduction over range, e.g., moving from 9 to 90 cm (100 unknows), which could be still acceptable considering the application, the computational time for each line of sight reduces to 0.7 s. Moreover, to improve the code performances, massive parallelization may be implemented (all lines of sight could be processed simultaneously). Both code optimization and parallelization could lead to two orders of magnitude speedup. For example, by employing a General Purpose Graphic Processor Unit (GP-GPU) with hundreds of cores, the global processing time can be further reduced. Such value could be further reduced by optimizing the code, making it suitable for most of real time applications.

## 4. Conclusions

Visual Driver Assistance Systems (VDAS) have a fundamental role in the automotive safety. However, VDAS are not reliable in poor visibility conditions. Imaging radars offer an effective alternative able to operate in any visibility conditions, in particular in critical situations due to the presence of smoke or fog. In this paper, we present a novel radar approach for signal processing in automotive field. In particular, a fast 2D focusing methodology based on the FFT algorithm followed by a new radar signal processing technique (based on Compressive Sensing) is presented. The proposed methodology is able to produce a 3D map of the scatterers within the imaged volume, providing the estimation both of their positions and reflectivities. Case studies have been presented in order to show the effectiveness of the approach and the performances achieved by the proposed algorithm. In particular, it has been shown that with respect to the widely adopted L^2^-norm minimization technique, the proposed methodology is able to estimate the reflectivity of multiple scatterers within the same LoS and their range distances in a more effective way, allowing strong subsampling of the available bandwidth. Reported results suggest that the proposed technique could be considered a promising and interesting approach for automotive radar focusing. In future work, the methodology will be tested and validated on other realistic and real datasets and studies on the detection and false alarm rates will be conducted.

## Figures and Tables

**Table 1:** Antenna characteristics.

Antenna Features	Values
Antenna type	Cross antenna
Central Frequency	77.25 GHz
Bandwidth	500 MHz
Gain	21.76 dB
Number of elements of antenna	111 × 141
Distance among the elements	1.9 mm (λ/2)
Antenna dimensions	21 × 27 cm

## Appendix

The signal model reported in Equation (1) can be written as:

(A1) $$S_{R}\left(x_{i},y_{j}\right)={∭}_{V}^{}\gamma \left(x,y,z\right)\frac{G}{{\left[R\left(x_{i}-x,y_{j}-y,z\right)\right]}^{2}}\cdot exp\left(i2\pi \frac{f_{0}}{c}\left(R_{T}+R_{R}\right)\right)dxdydz$$

where the distance 2*R* has been substitued with the sum of distances between the target, the transmitting antenna and the receiving one $\left(R_{T}+R_{R}\right)$. We want to focus the observed scenario at a fixed distance *z*_0_. In this case we can develop in a Taylor series the distances. By truncating at the 2nd order, we obtain:

(A2) $$R_{T}\approx z_{0}+\frac{x^{2}}{2z_{0}}+\frac{{\left(y^{\prime }-y\right)}^{2}}{2z_{0}},\;R_{T}\approx z_{0}+\frac{x^{2}}{2z_{0}}+\frac{{\left(y^{\prime }-y\right)}^{2}}{2z_{0}}$$

By substituting within Equation (A1), it becomes:

(A3) $$S_{R}\left(x^{\prime },y^{\prime },f\right)=\iint^{\text{​}}\gamma \left(x,y,d\right)\frac{G}{{\left[R\left(x^{\prime }-x.y^{\prime }-y,z_{0}\right)\right]}^{2}}\cdot exp\left(i\frac{2\pi }{\lambda }\left[2z_{0}+\frac{x^{2}}{2z_{0}}+\frac{{\left(y^{\prime }-y\right)}^{2}}{2z_{0}}+\frac{y^{2}}{2z_{0}}+\frac{{\left(x^{\prime }-x\right)}^{2}}{2z_{0}}\right]\right)dxdy=\iint^{\text{​}}\gamma \left(x,y,z_{0}\right)\frac{G}{{\left[R\left(x^{\prime }-x.y^{\prime }-y,z_{0}\right)\right]}^{2}}exp\left(i\theta \right)exp\left(i\frac{2\pi }{\lambda }\left[\frac{{\left(y^{\prime }-y\right)}^{2}}{2z_{0}}+\frac{{\left(x^{\prime }-x\right)}^{2}}{2z_{0}}\right]\right)dxdy$$

where we substituted  exp(iθ) = $exp\left(\frac{i2\pi }{\lambda }\left[2z_{0}+\frac{x^{2}+y^{2}}{2z_{0}}\right]\right).$

After the application of deramping, the received signal becomes, neglecting the amplitude terms:

(A4) $$S_{dr}\left(x^{\prime },y^{\prime },f\right)=S_{R}\left(x^{\prime },y^{\prime },f\right)\exp\left(-\frac{i2\pi \left(x^{2}+y^{2}\right)}{\lambda 2z_{0}}\right)=\iint^{\text{​}}\gamma \left(x,y,z_{0}\right)exp\left(i\phi \right)exp\left(-\frac{i2\pi }{\lambda }\frac{\left(xx^{\prime }+yy'\right)}{z_{0}}\right)$$

where exp(iϕ) = $exp\left(i\theta \right)exp\left(\frac{i2\pi }{\lambda }\frac{{x^{\prime }}^{2}+{y^{\prime }}^{2}}{2z_{0}}\right)$. By separating the *x* and *y* dependencies within the second exponential term, we obtain:

(A5) $$S_{dr}\left(x^{\prime },y^{\prime },f\right)=\iint^{\text{​}}\gamma \left(x,y,z_{0}\right)\exp\left(i\phi \right)exp\left(-\frac{i2\pi }{\lambda }\frac{xx'}{z_{0}}\right)exp\left(-\frac{i2\pi }{\lambda }\frac{yy'}{z_{0}}\right)dxdy$$

which recalls the 2D DFT kernel.
